# Factors influencing quality of life in Moroccan postmenopausal women with osteoporotic vertebral fracture assessed by ECOS 16 questionnaire

**DOI:** 10.1186/1477-7525-7-23

**Published:** 2009-03-13

**Authors:** Fatima E Abourazzak, Fadoua Allali, Samira Rostom, Ihsane Hmamouchi, Linda Ichchou, Laila El Mansouri, Loubna Bennani, Hamza Khazzani, Redouane Abouqal, Najia Hajjaj-Hassouni

**Affiliations:** 1 Department of Rheumatology, El Ayachi hospital, University Hospital of Rabat-Sale, Rabat, Morocco; 2 Laboratory of Information and Research on Bone Diseases (LIRPOS), Faculty of Medicine and Pharmacy, Rabat, Morocco; 3 Laboratory of Biostatistics, Clinical Research and Epidemiology (LBRCE), Faculty of Medicine and Pharmacy, Rabat, Morocco

## Abstract

**Objective:**

The aim of the study was to evaluate factors influencing quality of life (QOL) in Moroccan postmenopausal women with osteoporotic vertebral fracture assessed by the Arabic version of ECOS 16 questionnaire.

**Methods:**

357 postmenopausal women were included in this study. The participants underwent bone mineral density (BMD) measurements by DXA of the lumbar spine and the total hip as well as X-ray examination of the thoraco-lumbar spine to identify subclinical vertebral fractures. Patients were asked to complete a questionnaire on clinical and sociodemographic parameters, and osteoporosis risk factors. The Arabic version of the ECOS16 (Assessment of health related quality of life in osteoporosis questionnaire) was used to assess quality of life.

**Results:**

The mean age was 58 ± 7.8 years, and the mean BMI was 28.3 ± 4.8 kg/m^2^. One hundred and eight women (30.1%) were osteoporotic and 46.7% had vertebral fractures. Most were categorized as Grade1 (75%). Three independent factors were associated with a poor quality of life: low educational level (p = 0,01), vertebral fracture (p = 0,03), and history of peripheral fracture (p = 0,006). Worse QOL was observed in the group with vertebral fracture in all domains except "pain": Physical functioning (p = 0,002); Fear of illness (p = 0,001); and Psychosocial functioning (p = 0,007). The number of fractures was a determinant of a low QOL, as indicated by an increased score in physical functioning (p = 0,01), fear of illness (p = 0,007), and total score (p = 0,01) after adjusting on age and educational level. Patients with higher Genant score had low QOL in these two domains too (p = 0,002; p = 0,001 respectively), and in the total score (p = 0,01) after adjusting on age and educational level.

**Conclusion:**

Our current data showed that the quality of life assessed by the Arabic version of the ECOS 16 questionnaire is decreased in post menopausal women with prevalent vertebral fractures, with the increasing number and the severity of vertebral fractures.

## Background

Osteoporosis is a growing public health concern among the elderly population, particularly in postmenopausal women. It's a debilitating chronic disease that can reduce the quality of life (QOL) in a variety of ways, including diminished physical and emotional functioning. Vertebral fractures, the hallmark of osteoporosis, are commonly associated with back pain, kyphosis, and height loss. Therefore, they can lead to a reduced mobility and may be very painful, which can limit everyday activities [1,2]. Reduced activities can lead to increasing isolation, which, then, negatively impacts self-esteem and self-image, and causes depression. Studies have also shown that patients with vertebral fractures suffer from a loss of independence [3-5]. Anxiety and panic are reported early in osteoporosis [5]. All together, theses factors have an important impact on the quality of life of osteoporotic patients. Therefore, measuring the quality of life in post-menopausal women is important. Many questionnaires, either generic or disease-targeted, have been developed for the evaluation of QOL. Generic measures are applicable to various diseases, and even to the general population. Disease-targeted measures can include items that are more closely related to the disease process, and therefore can be more sensitive to the disease process when they are well designed.

Several specific questionnaires have been developed to measure QOL in osteoporosis. The most widely used are the Osteoporosis Quality of Life Questionnaire (OQLQ) [6,7], the Osteoporosis Assessment Questionnaire (OPAQ) [8-11], the Osteoporosis-Targeted Quality of Life Questionnaire (OPTQoL) [5,12-14], and the Quality of Life Questionnaire of the European Foundation for Osteoporosis (QUALEFFO) [6,15-21]. However their length and administration time have limited their use to clinical trials. For this reason, specific short form questionnaires, such as the mini-OQLQ [22] and the ECOS-16 (Assessment of health-related quality of life in osteoporosis) [23], have been developed.

There is no Arabic version of ECOS-16 to evaluate QOL in Moroccan osteoporotic women. QOL depends on the cultural background of each nation. Therefore, the QOL of Moroccan osteoporotic women should be evaluated using questionnaires developed for the Moroccan population.

The aim of this study was to assess QOL in Moroccan post-menopausal women with osteoporotic vertebral fractures using a standard Arabic version of ECOS-16.

## Patients and methods

### Patients

In this cross-sectional study, 357 ambulatory post-menopausal women living in urban areas of Morocco were sent to our outpatient Bone Densitometry Center. Recruitment was based on voluntary enrolment. All subjects were referred to this center for osteoporosis risk factors, including menopause. Informed consent was obtained from all subjects and the study was approved by ethics committee of our university hospital. We excluded from the study all patients with a history of: (1) taking drugs known to influence bone metabolism in the past 2 years, such as vitamin D, calcium, corticosteroids, bisphosphonates and hormone replacement therapy; (2) musculo skeletal, thyroid, parathyroid, adrenal, hepatic, or renal disease; (3) malignancy; and (4) hysterectomy. No adjunction or modification in treatment has been authorized.

### Data collection and measurements

Each patient completed a questionnaire on clinical and sociodemographic parameters, and osteoporosis risk factors. The age of menopause, the time since menopause, educational level, personal history of peripheral fracture, back pain, and comorbid conditions were recorded.

### Anthropometric data

Weight and height were measured without clothes or shoes at the time of bone densitometry measurements. The Body mass index (BMI) was calculated as body weight/height2 (kg/m2).

### Vertebral morphometry

Lateral radiographs of the thoracic and lumbar spine were made by standard methods. Morphometry was done from T4 to L4. Vertebral fractures were diagnosed by the Genant semiquantitative method [24], a visual radiographic approach which corresponds to the attribution of grades, ranging from 0 (no vertebral fracture); 1 (20% decrease of vertebra height); 2 (between 20 and 40% decrease of vertebra height); to 3 (severe vertebral fracture, more than 40% decrease of vertebra height). The severity of vertebral fractures was assessed by the Genant score.

### Bone mineral density (BMD) measurements

Lumbar spine, trochanter, femoral neck and total hip BMD were measured by dual-energy Xray absorptiometry with a Lunar prodigy densitometer. Daily quality control was carried out by measurement of a Lunar phantom. At the time of the study, phantom measurements showed stable results. The phantom precision expressed as the CV(%) was 0.08. Both T and Z scores were obtained. In the T-score calculations, the manufacturer's ranges for European reference population were used because of the absence of a Moroccan database. Osteoporosis was defined as a T-score lower than -2.5, according to the World Health Organisation study group definition [25].

### Quality of life evaluation: ECOS-16 Questionnaire

The specific QOL questionnaire: ECOS-16 was used to measure QOL. The 16 items are divided qualitatively into four dimensions: Pain; Physical functioning; Fear of illness; and Psychological functioning. ECOS-16 generates a single summary score obtained from the arithmetic mean of the answered items, so the total score ranges from 1 (best HRQOL) to 5 (worst HRQOL). The two summary scores PCS (Physical Component Summary) and MCS (Mental Component Summary) were also calculated.

The ECOS-16 questionnaire was adapted and translated into Arabic to be used in Moroccan patients with vertebral fractures. The translation followed proposed guidelines by Guillemin and et al [26,27]. In the first phase, the translation from the original language to the target language was done by two groups of translators. To ensure accuracy, the forward translation was back-translated into English by two other groups of translators with English culture totally blinded to the original version. The expert committee contained translators, back-translators, a sociologist, a teacher in linguistics, and two rheumatologists. Its role was to consolidate all the translated and back translated versions of the questionnaire, review the discrepancies, and develop the prefinal version of the questionnaire for field testing. A few questionable items were discussed and resolved. Globally, the adaptation did not cause any particular problems. Patients were asked to complete, the final Arabic version of ECOS-16, on 2 occasions separated by 1 week, to evaluate its reproducibility. For analphabet women, the questionnaire was read by third party without any modification of the content. Its acceptability was tested by studying the percentage of refusals, missing items, and complete questionnaires.

### Statistical analysis

Statistical analysis was performed with the Windows 13.0 version of SPSS software (SPSS Inc., Chicago, IL, USA). Values are expressed as mean ± S.D or percentages.

For the validation of the Arabic ECOS-16 questionnaire, internal consistency reliability was evaluated using Cronbach's alpha, and the test-retest reliability was evaluated by intra-class correlation coefficients (ICC) for the global score. Cronbach's alpha was calculated in each dimension of the instrument to assess the internal consistency reliability. A high alpha coefficient (≥ 0,70) suggests that the items within a dimension measures the same construct and supports the construct validity [28]. The ICC estimates the correlation between two measures among the same subject. Its value is comprised between +1 (perfect reproducibility) and 0 (hopeless reproducibility). A value above 0.80 is considered usually like satisfactory [29].

Item internal convergency represents the correlation between different domains. The domain which measures similar dimensions produces high correlations. Values above 0.60 correspond to a high correlation, moderate between 0.30 and 0.60, and low correlation below 0.30.

For the comparison between fractured and non-fractured patients, we used Student's t-test for quantitative variables and Chi-square test for qualitative variables. A logistic regression analysis was used to discriminate between the fractured and non fractured groups and to assess risk factors of vertebral fractures. Odds ratios (OR) and 95% confidence intervals (CI95%) were calculated.

In order to quantify the impact of the number and the severity of vertebral fractures on QOL, multiple linear regression was performed to assess independent factors associated with a poor QOL after adjusting on potential confounding variables.

A statistical significance level of p < 0.05 was used in all statistical tests performed.

## Results

### Study population

Table 1 shows the patients' sociodemographic and clinical characteristics with a comparison between the two groups according to the presence of vertebral fractures. The mean age of patients was 58.7 ± 7.8 years, and the mean of BMI was 28.3 ± 4.8. One hundred seventy two patients (48%) were housewives, and 68% were married. Of all participants, 27.4% were illiterate, 16% had received only primary school education, 38.7% secondary school, and 17.9% had been to high school. Overall, 46.5% reported at least one comorbid condition. Of all women, 30.1% were osteoporotic, and 46.7% had vertebral fractures. Most of them were determined to be Grade 1 (75%). The mean number of vertebral fractures was 2.4 ± 1.4. The majority was located at the thoracic level with 71 fractures (55.4%), 4 at the lumbar level (3%), and 51 (39.8%) at both thoracic and lumbar spine.

**Table 1:** Sociodemographic variables and clinical characteristics of menopausal women with and without vertebral fractures.

	**All patients** **(n = 357)**	**With vertebral fracture (n = 128)**	**Without vertebral fracture (n = 229)**	**p**
Age (years)	58.7 ± 7.8	61 ± 8.4	56.8 ± 6.7	<0.001

Age of menopause (years)	47.6 ± 5.3	46.9 ± 5.4	46.9 ± 7.5	0.89

Years since menopause: (years)	10.5 ± 9.7	14.6 ± 10	7.8 ± 8.5	<0.001

Marital status (%)				0.008
Married	68.1	60.2	73.6	
Single	3.4	1.6	4.7	
Widow	20.4	29.7	14.2	
Divorced	8.1	8.6	7.4	

Parity	3.7 ± 2.4	4.1 ± 2.6	3.4 ± 2.2	0.01

Education level (%)				0.002
No formal education	27.4	35.9	18.9	
Primary school	16	11.7	23	
Secondary school	38.7	32.8	43.2	
High school	17.9	19.5	14.9	

Body mass index (%)				0.7
≤ 30	66.6	67.2	64.9	
>30	33.4	32.8	35.1	

N° of comorbid conditions %)				0.11
None	51.6	42.3	54	
1–2	44	50.4	42.4	
≥ 3	4.4	7.3	3.6	

Non-vertebral fractures (%)				0.001
Presence	12	19.5	6.1	
Absence	88	80.5	93.9	

Back pain (%)				0.2
History	28.3	21.9	31.1	
Current	60.2	65.6	59.5	
Absence	11.5	12.5	9.5	

T-score				<0.001
Normal (%)	27.8	15.9	33.3	
Osteopenie (%)	42.1	34.9	51.4	
Osteoporosis (%)	30.1	49.2	15.3	

BMD				
Lumbar	0.977 ± 0.171	0.905 ± 0.175	1.016 ± 0.140	<0.001
Neck	0.852 ± 0.136	0.818 ± 0.130	0.873 ± 0.135	0.001
Trochanter	0.698 ± 0.123	0.655 ± 0.119	0.726 ± 0.111	<0.001
Ward	0.689 ± 0.153	0.635 ± 0.149	0.721 ± 0.137	<0.001
Femoral total	0.897 ± 0.136	0.838 ± 0.132	0.938 ± 0.117	<0.001

Continuous variables are expressed as mean ± SD

### Psychometric proprieties of the Arabic version of ECOS-16 questionnaire

The questionnaire had been generally well accepted by all patients. The mean duration of administration of the Arabic version of ECOS-16 was 5.8 ± 3.6 minutes. It has been correctly completed by 97% of patients with no missing or confusing items.

The internal consistency was very high with a Cronbach's alpha coefficient of 0.92 among the 16 items. Test-retest reliability was analysed with an Intraclass Correlation Coefficient of 0.92. When the different dimensions of ECOS-16 were analyzed, the internal consistency by parameter was good with a Cronbach's alpha coefficient comprised between 0.73 and 0.89 (Table 2).

**Table 2:** Internal consistency of the Arabic version of ECOS-16 questionnaire

	**Cronbach's alpha**
**Pain**	0.85
**Physical functioning**	0.79
**Fear of illness**	0.73
**Psychosocial functioning**	0.75
**Physical component summary (PCS)**	0.89
**Mental component summary (MCS)**	0.82
**Total score of ECOS-16**	0.92

All domains of ECOS 16 are correlated between themselves. The Spearman correlation coefficients are comprised between 0.328 and 0.756. The two dimensions of the ECOS 16 correlated significantly with each other (rho = 0.675) (Table 3).

**Table 3:** Correlation matrix of ECOS 16 questionnaire

	Pain	Physical functioning	Fear of illness	Psychosocial functioning	PCS	MCS	Total score
Pain	1.000						
Physical functioning	0.738	1.000					
Fear of illness	0.713	0.756	1.000				
Psychosocial functioning	0.328	0.520	0.586	1.000			
PCS	0.941	0.922	0.786	0.448	1.000		
MCS	0.565	0.704	0.868	0.910	0.675	1.000	
Total score	0.824	0.884	0.897	0.709	0.913	0.892	1.000

PCS = Physical Component Summary; MCS = Mental Component Summary

### Risk factors of vertebral fracture

In univariate analysis, vertebral fracture risk was significantly associated with older age (p < 0.001), with longer duration of menopause (p < 0.001), with higher parity (p = 0.01), with lower educational level (p = 0.002), with a history of peripheral fracture (p = 0.001), and with lower BMD at all the sites (p ≤ 0.001). Logistic regression showed that older age (OR = 1.05, CI95%: 1.01–1.09; p = 0,02), and lower lumbar BMD (OR = 0.02, CI95%: 0.01–0.13; p < 0,001) were independent factors of vertebral fracture after adjusting on age, educational level, history of peripheral fracture, and lumbar BMD.

### Factors associated with worse quality of life and the impact of vertebral fracture on quality of life

Univariate analysis showed that worse HRQoL was associated to older age (p < 0,001), higher BMI (p = 0,02), lower educational level (p ≤ 0,01), higher parity (p = 0,02), concomitant disease (p = 0,05), history of peripheral fracture (p < 0,001), and vertebral fracture (p = 0,003).

A multivariate analysis was carried out to identify patients' characteristics that were related to the ECOS-16 score. It shows that three independent factors were associated with a poor quality of life: low educational level (p < 0,05), vertebral fracture (p = 0,03), and a history of peripheral fracture (p = 0,006) (Table 4).

**Table 4:** Patients' characteristics influencing total ECOS-16 score in univariate and multivariate analysis.

	**Univariate analysis**			**Multivariate analysis**		
	***β***	***95% CI***	***p***	***β***	***95% CI***	***p***

**Age**	0.23	0.01 to 0.03	0.005	-0.003	-0.01 to -0.001	0.7
						
**BMI**	0.16	0.09 to 0.43	0.02	0.17	0.08 to 0.35	0.2
						
**Marital status**						
*Married*	*ref*			*ref*		
*Single*	0.16	0.04 to 0.28	0.7	0.06	0.02 to 0.07	0.3
*Widow*	0.47	0.01 to 0.62	0.2	0.44	0.01 to 0.67	0.07
*Divorced*	0.54	0.03 to 0.71	0.5	0.36	0.21 to 1.53	0.1
						
**Parity**	0.06	0.003 to 0.11	0.02	-0.02	-0.35 to -0.01	0.5
						
**Educational level**						
*No formal education*	*ref*			*ref.*		
*Primary school*	-0.74	-1.03 to – 0.45	< 0.001	-0.51	-0.82 to – 0.19	0.03
*Secondary school*	-0.64	-1.01 to – 0.45	0.01	-0.49	-0.85 to 0.12	0.009
*High school*	-0.30	-0.67 to – 0.12	0.01	-0.35	-0.40 to – 0.29	0.02
						
**Comorbid condition**	0.24	0.01 to 0.5	0.05	0.09	0.03 to1.13	0.5
						
**History of peripheral fracture**	0.65	0.30 to 0.99	< 0.001	0.54	0.11 to 0.92	0.006
						
**Vertebral fracture**	0.42	0.14 to 0.69	0.003	0.29	0.09 to0.52	0.03

Adjusting on age, BMI, marital status, parity, educational level, comorbid conditions, and history of peripheral fracture.

ref = Categorical of reference

Patients with at least one vertebral fracture had higher ECOS-16 scores in three domains (Table 5): Physical functioning (p = 0,002); Fear of illness (p = 0,001); Psychosocial functioning (p = 0,007), and in the two summary scores of ECOS-16: PCS (p = 0,01); MCS (p = 0,001).

**Table 5:** Values of the dimensions of the ECOS-16 in patients with and without vertebral fracture (VF)

	With VF Mean (SD)	Without VF Mean (SD)	p
Pain	2.89(1.20)	2.69 (1.03)	0.15
Physical functioning	2.33 (1.08)	1.96 (0.80)	0.002
Fear of illness	2.39 (0.96)	2.04 (0.72)	0.001
Psychosocial functioning	2.53 (1.15)	2.18 (0.93)	0.007
PCS	2.61 (1.09)	2.33 (0.82)	0.01
MCS	2.46 (0.95)	2.11 (0.72)	0.001

PCS = Physical Component Summary; MCS = Mental Component Summary

### Impact of the number of vertebral fracture on quality of life

Total score and all domains, except "pain", increased with increasing number of vertebral fractures in univariate analysis: Physical functioning (p < 0,001); Fear of illness (p < 0,001), psychosocial functioning (p = 0,008), and total ECOS-16 score (p = 0,001).

Linear regression shows that patients with higher number of fractures had worse QOL in two domains after adjusting on age and educational level: Physical functioning (p = 0,01); Fear of illness (p = 0,007), and total ECOS-16 score (p = 0,01) (Table 6).

**Table 6:** Impact of the number and the severity of vertebral fractures on QOL

	*Pain* *β(SE)*	*Physical function* *β(SE)*	*Fear of illness* *β(SE)*	*Psychosocial function* *β(SE)*	*Total score* *β(SE)*
Number of VF	0.04 (0.07)*	0.15 (0.06)**	0.15 (0.05)**	0.10 (0.07)*	0.19(0.07)**
Genant score	0.02 (0.02)*	0.05 (0.01)**	0.05 (0.01)***	0.02(0.02)*	0.05(0.02)**

*p: NS(>0.05); **p ≤ 0,01; ***p ≤ 0,001

Adjusting on age and educational level

VF: Vertebral fractures

### Severity of vertebral fractures and quality of life

The QOL was worse when the Genant score increased, as indicated by a higher score in different domains in univariate analysis: Physical functioning (p < 0,001); Fear of illness (p < 0,001); Psychosocial functioning (p = 0,009); total score (p = 0,01), and in multivariate analysis after adjusting on age and educational level: Physical functioning (p = 0,002), Fear of illness (p = 0,001), and total score (p = 0,01) (Table 6).

## Discussion

This study shows that vertebral fractures, their number and the severity of deformities have a negative impact on QOL. Indeed, ECOS-16 scores progressively increased in patients with vertebral fractures in all dimensions, except "Pain", and in both component summary scores (PCS and MCS). QOL was impaired in patients with greater number of vertebral fractures and higher Genant score except in the domains of "pain" and "psychosocial functioning". These findings underline the validity of the Arabic version of the ECOS 16 questionnaire.

We chose and used the ECOS-16 questionnaire because it is self-administered, short, simple and easy to score. Our study showed that cross-cultural adaptation of this questionnaire maintains the psychometric properties found in the original version. This was demonstrated through the short time needed to complete the questionnaire, the low percentage of incomplete questionnaires, the high alpha coefficients for internal consistency and the good reproducibility when using the test-retest. Cronbach's alpha was > 0.70 for the total score, the four dimensions, and the two summary scores of ECOS-16, which is in the range for internal consistency. Our results are similar to those reported in the original and Italian versions which has been validated recently for use in Italian patients [23,30].

All domains and the two summary scores correlated significantly between them (rho>0.3). The Italian study showed the same results [30].

Studies showing impaired QOL in patients with vertebral fractures have been published in other countries. Adachi and al [31], representing the Canadian Multicenter Osteoporosis Study (CaMos) Research Group, reported the association of fracture with lower QOL scores. As a part of the Multiple Outcomes of Raloxifene Evaluation (MORE) study, Oleksik and al [3] reported that patients with vertebral fractures had poorer scores on the QUALEFFO than those without vertebral fractures. These authors used three measures of QOL: the Nottingham Health Profile (NHP), the EQ-5D, and the QUALEFFO. Several studies have shown that HRQol progressively deteriorates in relation to the presence and number of vertebral fractures [32,33]. Badia and al reported the same result in the multivariate analysis [23]. Using the Italian version of ECOS-16, the presence and the number of vertebral fractures had also a negative effect on HRQoL (p < 0.001) [30]. In another study using QUALEFFO, the number and higher grade of fractures were determinant of a low QOL [34].

In our study, the domain of "pain" did not show differences either between patients with and without vertebral fractures or within patients according to the number and severity of vertebral fractures. Other studies found that the pain domain was discriminant in osteoporotic women, but patients were recruited on the basis of symptoms related to clinically apparent fractures and compared with patients without back pain [21,35]. This fact could be also explained because older fractures may be asymptomatic, or patients are already taking analgesics.

Another finding in the present study is that patient's educational level is also a determinant factor in the QOL impairment. In our study, a high level of education seems to be a protective factor against worse QOL. This finding has been reported in previous studies in patients with musculoskeletal problems [36,37], and was found in other versions of ECOS-16 [23,30]. It might be explained by the fact that women with higher levels of education tend to seek more information about their condition. This lead to better understanding of the disease and ability to cope with their condition through adherence to the prescribed medical regimen.

Our study has strengths and some limitations. The recruitment was not based on symptoms related to vertebral fractures. It had permit to evaluate the impact of old and recent, symptomatic and asymptomatic vertebral fractures on QOL. We also took into account comorbidities in the evaluation of the factors influencing the ECOS-16 score. Indeed, beside vertebral fractures, these comorbid conditions may influence QOL, especially in this elderly population. However, cross-sectional methodology did not allow us to compare the changes of QOL between patients with and without fractures. Moreover, the subjects were not recruited from the community at large, but rather, were selected from patients who underwent bone density determinations. This selection bias likely explains the relatively high prevalence of osteoporosis in the subjects studied.

## Conclusion

This study has revealed that QOL in Moroccan postmenopausal women is impaired by the presence of vertebral fracture, by the increasing number and by the severity of vertebral fractures. Currently, the endpoint in the treatment of osteoporosis is considered to be the prevention of fracture, with an increase of the BMD as the surrogate endpoint. Our data indicates that the measurement of QOL is mandatory for the evaluation of osteoporotic patients. This finding will not only provide an added parameter to evaluate the effectiveness of a given program, but will also focus care providers to be more attentive to the nonmedication aspects of osteoporosis management.

## Competing interests

The authors declare that they have no competing interests.

## Authors' contributions

FA, NHH and RA conceived the study and supervised its design, execution, and analysis and participated in the drafting and critical review of the manuscript. FA and RA did data management and statistical analyses. All other authors enrolled patients, participated in data acquisition and critical revision of the manuscript. FEA wrote the paper with input from all investigators.
